# Volatiles, A Glutarimide Alkaloid and Antimicrobial Effects of *Croton pullei* (Euphorbiaceae)

**DOI:** 10.3390/molecules18033195

**Published:** 2013-03-12

**Authors:** Rosana N. S. Peixoto, Giselle M. S. P. Guilhon, Maria das Graças B. Zoghbi, Isabella S. Araújo, Ana Paula T. Uetanabaro, Lourivaldo S. Santos, Davi do S. B. Brasil

**Affiliations:** 1 Programa de Pós-graduação em Química, Universidade Federal do Pará, Av. Augusto Corrêa 01, 66075-110, Belém-PA, Brazil; E-Mails: rosana.nsp@gmail.com (R.N.S.P.); lss@ufpa.br (L.S.S.); davibb@ufpa.br(D.S.B.B.); 2 Coordenação de Botânica, Museu Paraense Emílio Goeldi, CP 399, 66077-901, Belém-PA, Brazil; E-Mail: zoghbi@museu-goeldi.br; 3 Programa de Pós-graduação em Biotecnologia, Universidade Estadual de Feira de Santana, Av. Transnordestina s/n, 44036-900, Feira de Santana-BA, Brazil; E-Mail: araujo_isabella@yahoo.com.br; 4 Departamento de Ciências Biológicas, Universidade Estadual de Santa Cruz, Rodovia Ilhéus-Itabuna, s/n, 45662-900, Ilhéus-BA, Brazil; E-Mail: uetanabaro@yahoo.com

**Keywords:** *Croton pullei*, essential oils, glutarimide alkaloid, antibacterial activity, antifungal activity

## Abstract

Chemical investigation of *Croton pullei* (Euphorbiaceae) collected in the Brazilian Amazon region was revisited. The chemical composition of the essential oils of leaves and stems was analyzed by GC/MS. It was found that both the oils comprise mainly terpenes, among which linalool was the major one (24.90 and 39.72%, respectively). Phytochemical investigation of the stem methanol extract led to the isolation of a new natural product from the glutarimide alkaloid group named *N*-[2,6-dioxo-1-(2-phenylethyl)-3-piperidinyl]-acetamide, confirming that *C. pullei* is a rich source of this class of alkaloids. The hexane and methanol extracts of the stems of *C. pullei* showed moderate antibacterial and antifungal activity and the highest inhibition was observed when the methanol extract was tested against *Staphylococcus aureus* CCMB 262 and CCMB 263.

## 1. Introduction

**Figure 1:**
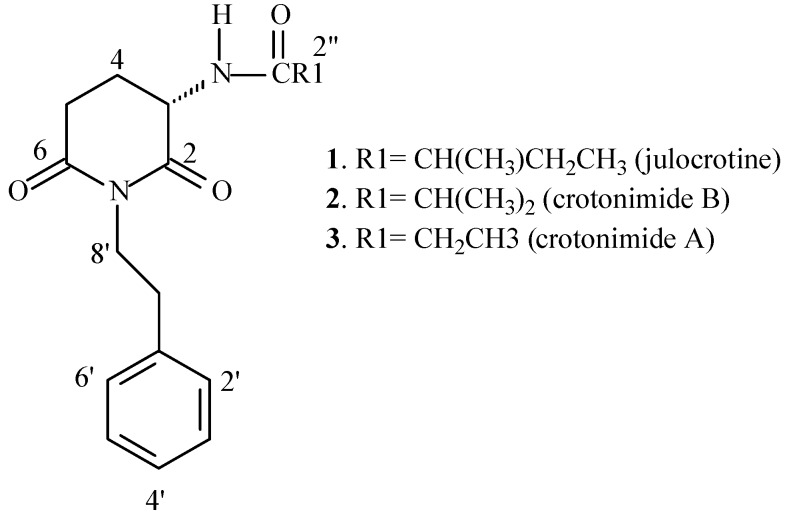
Structures of the glutarimide alkaloids from *Croton* *pullei*.

*Croton* L. is one of the largest genera of Euphorbiaceae, with about 1,200 species, mostly distributed in the West Indies and South America with some in North America [1,2], Africa and Madagascar [3]. Their species are trees, shrubs, herbs and lianas that occur in the most variable tropical ecosystems [4,5]. *Croton pullei* Lanj. is a small tree or a woody liana with restricted distribution, occurring in Guyana, French Guyana and Brazil (States of Pará and Maranhão) [5]. Some *Croton* species produce essential oils containing mostly terpenoids and phenylpropanoids, while some species produce only terpenoids [6]. Among the monoterpenes, α- and β-pinene, linalool and 1,8-cineole are often found; the most frequently found sesquiterpenes are β-caryophyllene and germacrene D, and among the phenylpropanoids, methyleugenol and related compounds are the most common; some other compounds, such as fatty acids, aliphatic esters and diterpenes are seldom cited [6]. Glutarimide alkaloids, among them julocrotine (**1**) and crotonimides B (**2**) and A (**3**) (Figure 1), terpenoids, flavonoids and a ferulamide derivative were previously isolated from *C. pullei* [7,8]. The structure of julocrotine was confirmed by X-ray analysis [9]. These glutarimide alkaloids were restricted to a small group of Euphorbiaceae, including *Julocroton* and some *Croton* species [10,11,12,13,14,15], but recently, julocrotine together with two other compounds of this class of substances were isolated from *Cordia* (Boraginaceae) [16]. Biological studies indicated that julocrotine has antiproliferative effects against the amastigote and promastigote forms of *Leshmania* (L.) *amazonensis* [17]. Several biological activities of *Croton* species extracts, essential oils and isolated compounds, including antibacterial and antifungal activities were reported [6]. A survey of the literature revealed that no studies on the volatile compounds or antimicrobial activity of *C. pullei* have been published to date. The aim of this study was to continue the chemical investigation of the non-volatile compounds of *C. pullei*, as it is a rich source of glutarimide alkaloids, and also to evaluate the chemical composition of the essential oil and the possible antimicrobial effects of this species.

## 2. Results and Discussion

### 2.1. Volatile Compounds

In total, 87 compounds were identified, accounting for 91–99.5% of the volatiles. The percentage of the compounds identified in the leaf and stem oils of *C. pullei* is listed along with their retention indices in Table 1. The yields of the essential oils from leaves and stems were 0.50% and 0.06%, respectively. The 57 compounds listed account for 87.20% and 91.95% of the volatiles of the leaves and stems, respectively. Except for the presence of 1-nonen-3-ol in the leaves and methyleugenol in the leaves and stems, terpenes made up the majority of the components, including monoterpenes (44.51% in leaves and 62.17% in stems) and sesquiterpenes (41.61% in leaves and 26.69% in stems). Oxygenated monoterpenes (42.56%) were major components in stem oil, while sesquiterpene hydrocarbons were predominant in the leaves. Similar amounts of monoterpene hydrocarbons and oxygenated sesquiterpenes were detected in both oils. The main compound identified in the leaf and stem oils was linalool (24.90 and 39.72%, respectively), followed by α-pinene (8.08 and 8.23%, respectively) and β-pinene (6.63 and 9.81%, respectively). Comparing the chemical composition of the two oils, it is clear that there are only quantitative differences between the major compounds. On the other hand, the two oils showed a great qualitative difference, with the presence of twenty-three compounds detected only in the leaf oil, and fourteen in the stem oil.

**Table 1:** Volatiles (%) identified in the leaf and stem oils of *Croton pullei*.

Constituents	RI *	Leaf	Stem
α-pinene	937	8.08	8.23
β-pinene	981	6.63	9.81
Myrcene	994		1.07
1,8-cineole	1033	3.72	
γ-terpinene	1060		0.50
*cis*-linalool furanoxide	1074	0.26	
1-nonen-3-ol	1080	0.60	0.71
*trans*-linalool furanoxide	1091	0.32	
Linalool	1105	24.90	39.72
α-campholenal	1129		0.67
α-terpineol	1193	0.60	
*trans*-pinocarveol	1142		0.49
*trans*-sabinol	1148		0.40
terpinen-4-ol	1180		0.68
Myrtenal	1200		0.60
β-elemene	1395		1.24
γ-elemene	1340	0.96	
α-cubebene	1352	0.36	
α-copaene	1379	0.31	
β-elemene	1388	0.44	
Methyleugenol	1408	0.48	0.45
*cis*-α-bergamotene	1419	0.30	
β-caryophyllene	1424	3.96	1.24
*trans*-α-bergamotene	1439		0.27
γ-elemene	1437	0.73	
Aromadendrene	1444	0.15	
α-humulene	1459	1.84	
*allo*-aromadendrene	1466	0.29	
γ-gurjunene	1481	0.90	0.43
germacrene D	1486	1.19	
β-selinene	1492	2.91	1.25
α-selinene	1500	2.67	1.21
bicyclogermacrene	1502	2.67	
α-muurolene	1505		0.15
(*Z*)-α-bisabolene	1510	0.46	
γ-cadinene	1519	1.50	
δ-cadinene	1527	1.12	0.78
*cis*-calamenene	1537	1.15	0.69
*trans*-cadina-1,4-diene	1545	0.08	
α-calacorene	1545	0.08	0.45
(*E*)-nerolidol	1564	3.91	4.62
Spathulenol	1580	2.72	2.38
caryophyllene oxide	1585	1.80	1.71
Viridiflorol	1604	0.16	
Ledol	1616	0.78	
Junenol	1625		1.00
*iso*-spathulenol	1631	0.40	
*epi*-cadinol	1642	0.57	
α-muurolol	1650	0.57	
Pogostol	1660	3.58	4.65
(*Z*)-α-santalol	1685	1.63	2.05
Acorenone	1697	0.53	1.13
(*Z*)-*epi*-β-santalol	1703	0.80	0.57
cyclocolorenone	1759	0.09	0.87
manoyl oxide	2002		0.87
13-*epi*-manoyl oxide	2025		0.39
2-oxo-manoyl oxide	2243		0.68

* RI on Rtx-5MS.

Despite the occurrence of linalool in the leaves and stems of *C. pullei*, their oxides were detected only in the leaf oil. *Croton* species containing high amounts of linalool were *C. cajucara* Benth. [18], *C. lanjouwensis* Jabl. [19], *C. aubrevillei* J. Léonard [20] and *C. micradenus* Urb. [21]. High amounts of α-pinene were found in some *Croton* species, such as *C. adenocalyx* Baill. [22], *C. antanosiensis* Leandri [23], *C. matourensis* Aubl. [24] and *C. micradenus* Urb. [21]. This is the first report on the chemical composition of the essential oils of these species.

### 2.2. Novel Non-Volatile Compound

**Figure 2:**
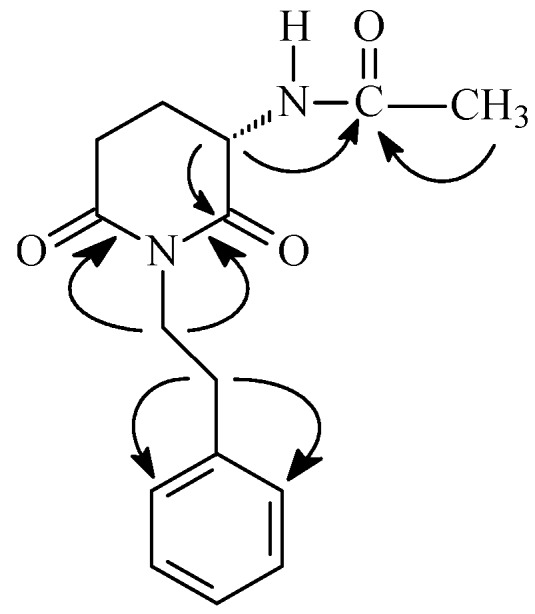
Structure and correlations of the HMBC spectrum of substance **4**.

Substance **4** was isolated from the methanol extract of the stems of *C. pullei* and identified as the glutarimide alkaloid *N*-[2,6-dioxo-1-(2-phenylethyl)-3-piperidinyl]-acetamide (Figure 2). This is the first time that compound **4** is isolated as a natural product; until now it has only been obtained by synthesis [25]. It was isolated as a light brown amorphous solid, soluble in methanol, with ${\left[\alpha \right]}_{\text{D}}^{25}$ = −11° (*c* 0.03, CH_3_OH) (${\left[\alpha \right]}_{\text{D}}^{20}$ −8.2° in CHCl_3_ [25]) and IR absorptions at 3333, 2901, 2852, 1726, 1678, 746, 695 cm^−1^. The molecular formula of substance **4** was suggested to be C_15_H_18_O_3_N_2_ from the HRMS with M^+^ 274.1317 Da (calculated 274.131743). The ^1^H-NMR spectrum (CD_3_OD) displayed signals of a phenylethyl group (H-2'-H-8') similar to those of julocrotine and crotonimides A and B [7] and signals of a glutarimide unit with the H-3 signal unfolding in a double doublet at *δ*_H_ 4.60 (*J* = 12.6 and 5.6 Hz) and the H-4 and H-5 signals in multiplets at *δ*_H_ 2.13–1.86 and 2.75–2.71, respectively; the signal of hydrogen in the group N-H was not observed or it was rather weak and the signal of the acetyl side chain was observed as a singlet at *δ*_H_ 2.02. The ^13^C-NMR spectrum (CD_3_OD) showed the signals of a phenylethyl and glutarimide moieties and of an *N*-acetyl group with three carbonyl signals at *δ*_C_ 173.3, 173.4 and 172.9. All assignments were based on 2D-NMR data of ^1^H-^1^H COSY (300 MHz), HETCOR (^13^C-^1^H COSY, ^1^*J*(CH), 75 MHz), HMBC [^1^H-^13^C COSY, ^2,3^*J*(CH)] experiments. Figure 2 shows the most important HMBC correlations of substance **4**. The NMR data of **4** reported before were partial and recorded in CDCl_3_ and those reported here were recorded in CD_3_OD and are now fully assigned (Table 2).

**Table 2:** ^1^H (300 MHz) and ^13^C (75.5 MHz) NMR data of substance **4** (CD_3_OD).

	*δ*_H_	*δ*_C_
2	-	173.3 *
3	4.60 (*dd*, *J* = 12.6 and 5.6 Hz)	51.8
4	2.13–1.86 (*m*)	24.8
5	2.75–2.71 (*m*)	32.4
6	-	172.9 *
1'	-	139.9
2'	7.29–7.18 (*m*)	129.9
3'	7.29–7.18 (*m*)	129.5
4'	7.29–7.18 (*m*)	127.3
5'	7.29–7.18 (*m*)	129.5
6'	7.29–7.18 (*m*)	129.9
7'	2.77 (*t*, *J* =7.8 Hz)	34.8
8'	3.95–3.80 (*m*)	42.6
1''	-	173.7
2''	2.02 (*s*)	22.5

* Signals can be interchanged.

### 2.3. Antimicrobial Activity

The methanol and hexane extracts of *C. pullei* were evaluated for their antimicrobial activity against Gram-negative bacteria, Gram-positive bacteria and yeast species responsible for various forms of acquired infections in humans and, more often, antimicrobial resistance. The MIC and MCC data on the hexane and methanol extracts obtained from the stems of *C. pullei* are shown in Table 3.

**Table 3:** Results of Minimum Inhibitory Concentration (MIC) and Minimum Microbicidal Concentration (MMC) in mg mL^−1^, of the hexane and methanol extracts of *C.* *pullei* stems.

**Organism**	**Hexane**		**MeOH**		**Control**	
	MIC	MMC	MIC	MMC	Nist/Chlorf	DMSO
*Escherichia coli* CCMB 261	5	-	5	5	R	5.00
*Pseudomonas aeruginosa* CCMB 268	5	10	1.25	2.5	0.31	5.00
*Salmonella* sp. CCMB 281	2.5	10	1.25	10	0.16	5.00
*Staphylococcus aureus* CCMB 262	0.156	5	0.078	2.5	0.31	5.00
*S. aureus* CCMB 263	1.25	10	0.078	5	0.31	10.00
*S. aureus* CCMB 285	10	10	2.5	5	R	10.00
*Bacillus cereus* CCMB 282	2.5	2.5	1.25	1.25	0.16	5.00
*Candida albicans* CCMB 286	2.5	5	2.5	5	0.63	10.00
*C. albicans* CCMB 266	2.5	10	2.5	5	0.08	10.00
*C. parapsilosis* CCMB 288	2.5	10	2.5	10	R	10.00

R: resistent, Nyst: nystatin, Chlorf: chloramphenicol.

Both extracts showed inhibition of the tested microorganisms and the effect of the methanol extract was higher than the hexane extract. The highest inhibition effect of the methanol extract was observed against the Gram-negative bacterium *Pseudomonas aeruginosa*. CCMB 268 (MIC = 1.25 mg mL^−1^), and the Gram-positive bacteria *S. aureus* CCMB 262 (MIC = 0.078 mg mL^−1^), *S. aureus* CCMB 263 (MIC = 0.078 mg mL^−1^) and *B. cereus* CCMB 282 (MIC = 1.25 mg mL^−1^). The hexane extract was more active against *S. aureus* CCMB 262 (MIC = 0.156 mg mL^−1^) and *S. aureus* CCMB 263 (MIC = 1.25 mg mL^−1^).

According to Fontanay *et al.* [26] MIC values below 10 μg mL^−1^ are considered good and those around 50 μg mL^−1^ moderate for antibacterial activity. MIC values that equal hundreds of μg mL^−1^ indicate that the compound has no activity. Thus, it can be considered that the methanol extract of *C. pullei* has moderate activity against both *S. aureus* tested strains.

In similar a study, Selowa and coworkers [27] using extracts of three *Croton* species (*C.* *megalobotrys*, *C.* *steenkampianus* and *C.* *salvaticus*) observed that the methanol extract of *C.* *megalobotrys* was the most active extract inhibiting *S.* *aureus* at 0.625 mg mL^−1^, *P.* *aeruginosa* at 0.313 mg mL^−1^ against and *E.* *coli* at 0.125 mg mL^−1^. The methanol extracts of *C. campestris* [28] and *C. membranaceus* showed antimicrobial activity against *S. aureus*.

It is known that the stem extracts of *C. pullei* are a rich source of glutarimide alkaloids, but despite the high amounts of julocrotine (**1**) in this species, it seems that this compound does not contribute to the antimicrobial activity of the test extracts, since according to Bayor and coworkers julocrotine exhibited no significant activity against *S. aureus*, *Bacillu subtilis* and *P. aeruginosa* [29] and that’s why it was not tested again. On the other hand, among the isolated compounds from *C. pullei* in prior chemical investigations [7], the diterpenes kaurenoic acid and ribenone and the triterpene lupeol are known for their antimicrobial activities [30,31,32,33] and could contribute for the observed activity of the extracts.

## 3. Experimental

### 3.1. Material and Isolation and Identification of Non-Volatile and Distillation of the Volatile Constituents

Samples of *C. pullei* were taken from the wild plant in a secondary forest in the municipality of Peixe-Boi, State of Pará, Brazil (October, 2008). A voucher specimen (# 188,908) was kept in the Herbarium MG of the Museu Paraense Emílio Goeldi (MPEG).

### 3.2. Extraction of Volatile Compounds

The samples were dried for 7 days in an air-conditioned room (at low humidity) and then ground. Leaves (100 g) and stems (80 g) were hydrodistilled for 3 h using a Clevenger-type apparatus with the refrigeration water maintained at 15 °C. The oils obtained were centrifuged for 5 min (3,000 rpm), dried over Na_2_SO_4_, centrifuged again, and immediately submitted to GC/FID and GC/MS analysis. The solution containing 2 µL of the oil in 1 mL of hexane was immediately prepared to gas chromatography analysis. The total oil yield was expressed in percentage (volume/mass) on the basis of dried material.

### 3.3. Analysis of the Volatiles

The oils were analyzed using a Shimadzu GC/MS Model QP 2010 Plus, equipped with a Rtx-5MS (30 m × 0.25 mm; 0.25 μm film thickness) fused silica capillary column. Helium was used as carrier gas adjusted to 1.2 mL.min^−1^; with splitless injection of 1 μL of a hexane solution; injector and interface temperature were 250 °C; oven temperature programmed was 60–240 °C at 3 °C.min^−1^. EIMS: electron energy, 70 eV; ion source temperature was 200 °C. Identification of the compounds were made by comparison of their GC mass and retention data with those in NIST-05 library and cited in the literature data [34]. Retention indices were calculated using *n*-alkane standard solutions (C8-C26) available from Fluka S. A. (Steinheim, Switzerland), in the same chromatographic conditions. Quantitative data were obtained from the electronic integration of the total ion chromatogram (TIC) peak areas.

### 3.4. Isolation and Identification of Compound ***4***

The stems of *C. pullei* were extracted as in [8]. The dichloromethane phase of the methanol extract was fractionated by column chromatography on silica using mixtures of hexane, ethyl acetate and methanol in gradients of increasing polarities as eluents. The fraction eluted with hexane-EtOAc 70% was purified by column chromatography on Sephadex LH-20 using methanol as eluent leading to the isolation of 41 mg of compound **4**. Spectrometric methods were used for structural determination. NMR spectra were recorded on a Varian 300 MHz NMR spectrometer (300 MHz and 75 MHz for ^1^H and ^13^C, respectively) using TMS as internal standard; the IR spectrum was recorded on a Thermo electron IR 100 spectrometer; optical rotation was measured at the sodium D line (589 nm) on a Perkin Elmer 341 and the HRMS was recorded on a VG Auto Spec-300.

### 3.5. Antimicrobial Assays

#### 3.5.1. Microbial Strains

The following bacteria and yeasts were used for the experiments: *Escherichia coli* CCMB 261 (sensitive to trimetoprime and resistant to sulphonamide), *Pseudomonas aeruginosa* CCMB 268, *Salmonella* sp. CCMB 281, *Staphylococcus aureus* CCMB 262 (resistant to streptomycin and dihydrostreptomycin), *Staphylococcus aureus* CCMB 263, *Staphylococcus aureus* CCMB 285, *Bacillus cereus* CCMB 282, *Candida albicans* CCMB 286, *Candida albicans* CCMB 266 and *Candida parapsilosis* CCMB 288 (resistant to amphoterycin-B). All microorganisms were cultured on Müeller-Hinton agar (MHA). The bacterial strains were cultured at 37 °C for 24h and yeasts at 28 °C for 48h. All the microbial tests were performed in triplicate.

#### 3.5.2. Minimum Inhibitory Concentration (MIC)

The minimum inhibitory concentration (MIC) of the methanol extract of the leaves of *C. pullei* was determined based on a micro dilution method in a 96 multi-well microtiter plates [35]. All microbial tests were performed in MHA. The extracts were dissolved DMSO-water solution (1:1) and sterilized by filtration through cellulose acetate membrane (0.22 mm). Serial dilutions from 10 to 0.078 10 to 0.078 mg mL^−1^ of the extracts were prepared. Each well received 10 μL of suspension of each micro-test. The purity of the suspension of the inoculums was verified in a simultaneous incubation. After the period of incubation, 50 μL of triphenyltetrazolium chloride 2-3-5 (TTC) was added to a final concentration of 0.40 mg mL^−1^ (final concentration; assays with yeasts) and 30 μL of rezasurine (RZ, assays with bacteria) to a final concentration of 0.01% for qualitative analysis of microbial growth in the wells in order to determine the antimicrobial activity of each dilution of the samples. Nistatine (20 mg mL^−1^) and cloramphenicol (10 mg mL^−1^) were used as positive controls. Controls were performed to test the viability of microorganisms and the sterility of the culture medium. The MIC was considered the lowest extract concentration where there was no visible microbial growth after color indicator (TTC and RZ) step.

#### 3.5.3. Minimal Microbicidal Concentration (MMC)

Petri dishes containing MHA were used for this assay; 5 µL of each MIC well were transferred to MHA and cultured at 28 °C for 48 h (yeasts) and at 37 °C for 24 h (bacteria). The MMC was considered the lowest extract concentration where there was no cellular growth.

## 4. Conclusions

The essential oils of the leaves and stems of *Croton pullei* were predominantly composed of terpenes, and the major constituent of both oils (linalool) showed only small quantitative variations (24.90 and 39.72%, respectively). The isolation of the new natural product substance **1** confirms the ability of this species to produce glutarimide alkaloids. The hexane and methanol extracts of the stems of *C. pullei* showed moderate antibacterial and antifungal activity and the highest inhibition was observed against *Staphylococcus aureus* CCMB 262 and CCMB 263 for both extracts. The presence of kaurenoic acid, ribenone and lupeol in the stems extracts of *C. pullei*, but not julocrotine, can explain in part the observed antimicrobial activity.
